# LatAm‐FINGERS: Navigating Diversity in the Engagement of a Subcontinental Trial for Dementia Prevention

**DOI:** 10.1002/alz.086708

**Published:** 2025-01-09

**Authors:** Ismael Luis Calandri, Lucia Crivelli, Paulo Caramelli, Francisco Lopera, Ricardo Nitrini, Ana Luisa Sosa, Lina Marcela Vellila, Rosa Maria Salinas‐Contreras, Claudia Kimie Suemoto, Monica Sanches Yassuda, Gustavo Sevlever, Daisy M Acosta, Ana Charamelo, María Isabel Cusicanqui, Nilton Custodio, Carolina Delgado, Lissette Duque, Ivonne Z. Jimenez Velazquez, Jorge M Leon‐Salas, Heather M Snyder, Maria C. Carrillo, Miia Kivipelto, Ricardo Allegri, LatAm‐FINGERS Consortium

**Affiliations:** ^1^ Fleni, Buenos Aires, Buenos Aires Argentina; ^2^ Fleni, Buenos Aires, CABA Argentina; ^3^ Faculty of Medicine ‐ Universidade Federal de Minas Gerais, Belo Horizonte Brazil; ^4^ Grupo de Neurociencias de Antioquia, Facultad de Medicina, Universidad de Antioquia, Medellín Colombia; ^5^ Cognitive and Behavioral Neurology Unit ‐ University of São Paulo, São Paulo Brazil; ^6^ Dementias Laboratory, National Institute of Neurology and Neurosurgery, Mexico City, DF Mexico; ^7^ Grupo de Neurociencias de Antioquia, Universidad de Antioquia, Medellin Colombia; ^8^ Division of Geriatrics, Department of Internal Medicine, University of Sao Paulo Medical School, São Paulo, São Paulo Brazil; ^9^ University of São Paulo, SAO PAULO, SAO PAULO Brazil; ^10^ Universidad Nacional Pedro Henriquez Ureña, Santo Domingo, Distrito Nacional Dominican Republic; ^11^ Hospital Británico, Montevideo, Montevideo Uruguay; ^12^ Departamento de Neuropsicología, Facultad de Medicina‐Hospital de Clínicas, Universidad de la República, Montevideo Uruguay; ^13^ Centro Neurológico Mente Activa, La Paz Bolivia (Plurinational State of); ^14^ Instituto Peruano de Neurociencias, Lima, Lima Peru; ^15^ Universidad de Chile, Santiago, Region Metropolitana Chile; ^16^ Neuromedicenter, Quito Ecuador; ^17^ University of Puerto Rico, School of Medicine, San Juan, Puerto Rico, PR USA; ^18^ Clínica Bíblica Hospital, San José Costa Rica; ^19^ Alzheimer’s Association, Chicago, IL USA; ^20^ Department of Neurobiology, Care Sciences and Society, Division of Clinical Geriatrics, Center for Alzheimer Research, Karolinska Institutet, Stockholm/Solna Sweden; ^21^ Population Health Unit, Finnish Institute for Health and Welfare, Helsinki Finland; ^22^ The Ageing Epidemiology (AGE) Research Unit, School of Public Health, Imperial College London, London United Kingdom

## Abstract

**Background:**

LatAm‐FINGERS is the first non‐pharmacological multicenter randomized clinical trial aimed at preventing cognitive impairment in Latin America. It encompasses twelve countries that collectively represent 45% of the territory in the Americas. Its objective is to reach populations that, despite sharing commonalities such as language, are culturally and economically diverse. Above all, these populations have different perspectives on goals related to healthy aging, access to healthcare, and participation in clinical trials. The challenge is twofold: on one hand, achieving commitment across all centers to ensure recruitment and adherence; on the other hand, utilizing these strategies to instill awareness in society about the scourge of dementia and the possibility of its prevention.

**Methods:**

Our aim is to describe the recruitment strategies and their effectiveness in the design and implementation of the initial phases of the trial. The most relevant strategies included: a) decentralized design, where the 12 site principal investigators collaborated across all phases of the trial design rather than merely implementing it. They were asked to present solutions and barriers based on the idiosyncrasies of their target population and share them with the rest. b) assess cultural beliefs about clinical trials, c) disseminate information about dementia prevention strategies, d) collaborate with community partners in outreach and recruitment.

**Results:**

The most utilized strategies included 1) assess population with cognitive complaints or concern for prevention (for centers with clinical setting) and 2) community dissemination for universities and research centers. A total of 1,877 candidates were evaluated, of which 1,232 were included. Pre‐screening strategies were implemented to reduce staff burden at most centers.

**Conclusions:**

Our results demonstrate that, despite the diversity present in Latin America, it is possible to conduct a unified trial with successful recruitment. Carefully designed strategies that respect local culture, coupled with knowledge of local stakeholders, hold the key to success. Moreover, it highlights that a clinical trial, in addition to being a tool to test treatment effectiveness, can serve as a catalyst for awareness campaigns.

